# Electrical stimulation therapy for peripheral nerve injury

**DOI:** 10.3389/fneur.2023.1081458

**Published:** 2023-02-24

**Authors:** Lingmei Ni, Zhao Yao, Yifan Zhao, Tianfang Zhang, Jie Wang, Siyue Li, Zuobing Chen

**Affiliations:** ^1^Infection Prevention and Control Department, The First Affiliated Hospital, School of Medicine, Zhejiang University, Hangzhou, China; ^2^The Second Affiliated Hospital and Yuying Children's Hospital, Wenzhou Medical University, Wenzhou, China; ^3^Department of Rehabilitation Medicine, The First Affiliated Hospital, School of Medicine, Zhejiang University, Hangzhou, China

**Keywords:** peripheral nerve injury, peripheral nerve regeneration, electrical stimulation (ES), therapy, mechanism

## Abstract

Peripheral nerve injury is common and frequently occurs in extremity trauma patients. The motor and sensory impairment caused by the injury will affect patients' daily life and social work. Surgical therapeutic approaches don't assure functional recovery, which may lead to neuronal atrophy and hinder accelerated regeneration. Rehabilitation is a necessary stage for patients to recover better. A meaningful role in non-pharmacological intervention is played by rehabilitation, through individualized electrical stimulation therapy. Clinical studies have shown that electrical stimulation enhances axon growth during nerve repair and accelerates sensorimotor recovery. According to different effects and parameters, electrical stimulation can be divided into neuromuscular, transcutaneous, and functional electrical stimulation. The therapeutic mechanism of electrical stimulation may be to reduce muscle atrophy and promote muscle reinnervation by increasing the expression of structural protective proteins and neurotrophic factors. Meanwhile, it can modulate sensory feedback and reduce neuralgia by inhibiting the descending pathway. However, there are not many summary clinical application parameters of electrical stimulation, and the long-term effectiveness and safety also need to be further explored. This article aims to explore application methodologies for effective electrical stimulation in the rehabilitation of peripheral nerve injury, with simultaneous consideration for fundamental principles of electrical stimulation and the latest technology. The highlight of this paper is to identify the most appropriate stimulation parameters (frequency, intensity, duration) to achieve efficacious electrical stimulation in the rehabilitation of peripheral nerve injury.

## 1. Introduction

The peripheral nervous system, including cranial nerves, spinal nerves, and autonomic nerves, is a nerve trunk, nerve plexus, ganglion, and nerve terminal composed of perikarya and nerve fibers, which mainly connect the peripheral sensory apparatus and the central nervous system. Peripheral nerve injury (PNI) is a kind of motor and sensory disorder caused by damage to the structure of peripheral nerves. The incidence of PNI caused by trauma is roughly 5%, including brachial plexus and root injuries (1). After nerve injury, damaged axons are not able to regenerate completely. Therefore, it is important to provide appropriate therapies to reconnect nerves in the injured area and to accelerate the growth rate of nerve (2). At present, the treatment methods for nerve injury are mainly divided into surgical treatment and non-surgical treatment (3–5). Electrical stimulation (ES) is the most commonly used non-surgical treatment. Most studies used low-frequency ES to promote nerve regeneration, but the method and frequency range of ES need to be standardized because high-frequency ES will aggravate nerve damage (6). The standard ES parameter of 20 Hz for one hour immediately after the repair is well known. However, its utility and efficacy for various nerves have never been defined. This review focuses on a framework to develop a new ES paradigm enabling future clinical translation.

## 2. Peripheral nerve anatomy

Peripheral nerves refer to all nerves other than the brain and spinal cord, including ganglia, nerve trunks, nerve plexus and nerve ending. The center of the anatomical structure of the peripheral nerve is the nerve fiber. The endoneurium wraps around the nerve fibers to form the nerve bundle that is surrounded by loose connective tissue. The epineurium then wraps the nerve bundle to form a complete peripheral nerve (7). There are many pathogenic factors, such as infection, ischemia, trauma, metabolic disorders, poisoning, nutritional deficiency, and iatrogenic injury (such as chemotherapy, radiotherapy, etc.). Peripheral injury can lead to severe dysfunction, often affecting the ability of patients to perform activities of daily living.

## 3. Classification of peripheral nerve injury

**Figure 1:**
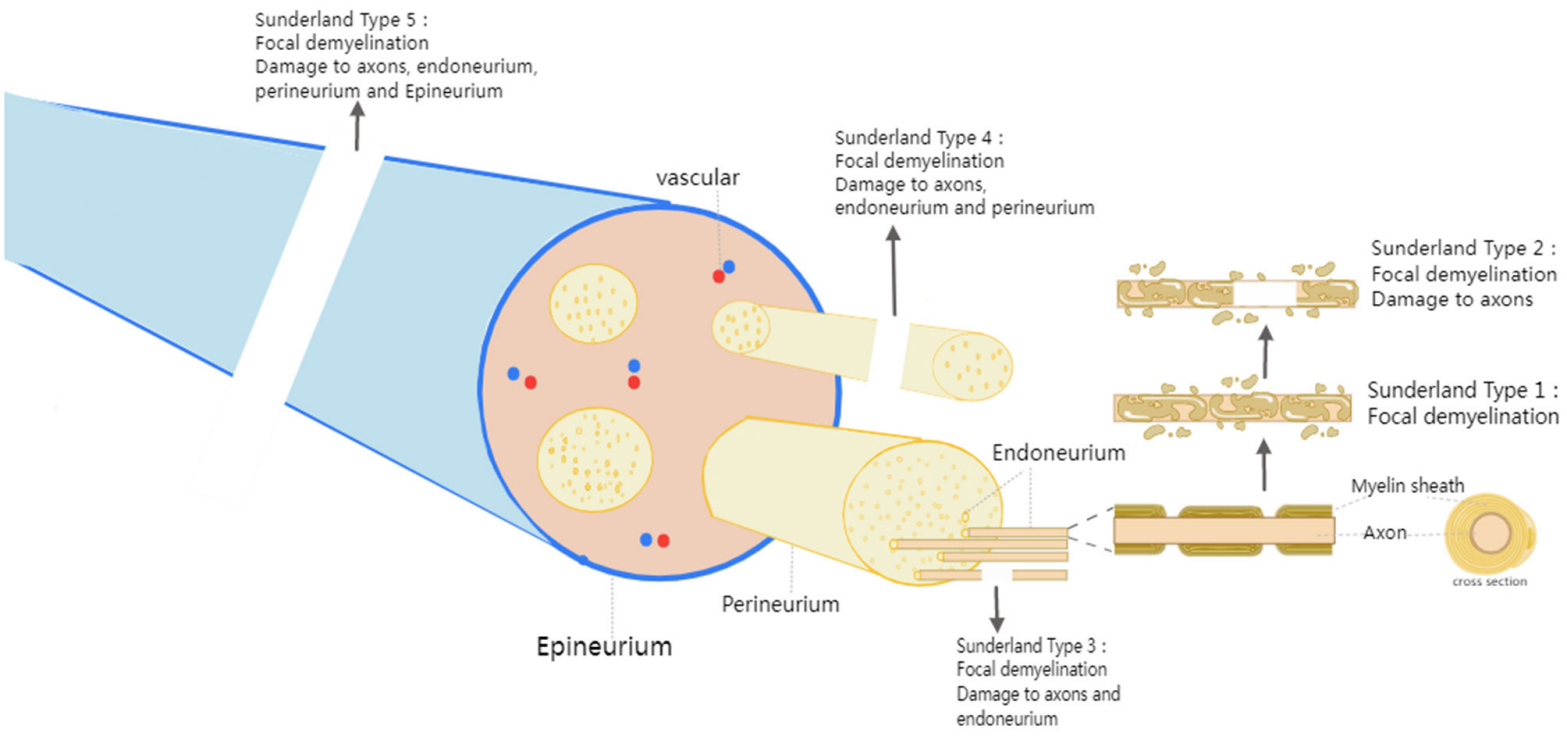
Peripheral nerve anatomy and classification of peripheral nerve injuries.

Common peripheral nerve injuries include brachial plexus nerve injury, axillary nerve injury, cutaneous nerve injury, median nerve injury, radial nerve injury, ulnar nerve injury, femoral nerve injury, sciatic nerve injury, and common peroneal nerve injury. According to Seddon's classification in 1943, PNI was divided into three types neurapraxia, axonal disruption, and nerve rupture. According to the 1951 Sunderland classification, the PNI was divided into 5 types. Type I: focal demyelination; Type II: damage to axons but endoneurium, perineurium, and epineurium intact; Type III damage to axons and endoneurium but perineurium and epineurium intact; Type IV: damage to axons, endoneurium, and perineurium but epineurium intact; Type V: complete loss of continuity (Figure 1). For non-severe injuries (Types I-III), Patients only required exercise training and physical therapy as treatment because adult nerves have some intrinsic regenerative capacity. For type IV and V injuries, it is necessary to suture the nerve transection by surgeries, including nerve manipulation and bridging (8).

## 4. Treatments of electrical stimulation

Currently, the surgical treatment of PNI includes microsurgical end-to-end repair, tension-free nerve epineurium suture, and autologous nerve grafting (9, 10). Less than half of patients recover satisfactory motor and sensory function after nerve repair. A third of patients have little or no recovery despite proper surgery.

Although great advances have been made in surgical strategies to treat PNI, most patients did not undergo systematic rehabilitation after surgery, leaving them with lifelong sensorimotor disorders and chronic neuropathic pain. There is a need for a treatment to overcome the limitations of peripheral nerve recovery and improve patient physical function. ES is a promising method to accelerate peripheral nerve regeneration. After PNI, ES has been shown to promote early stages of nerve regeneration, including neuronal survival and axon bud formation (11).

ES can not only treat PNI but also show the changes in the process of injury. The latest research has shown that the use of pulsed ES to act on the injured muscle and record the changes in muscle fibers, called the muscle velocity recovery cycle (MVRC), can provide a detailed understanding of the in vivo evidence of depolarized resting potential after PNI. To provide the reason for neurogenic muscle weakness caused by reduced muscle excitability (12).

**Figure 2:**
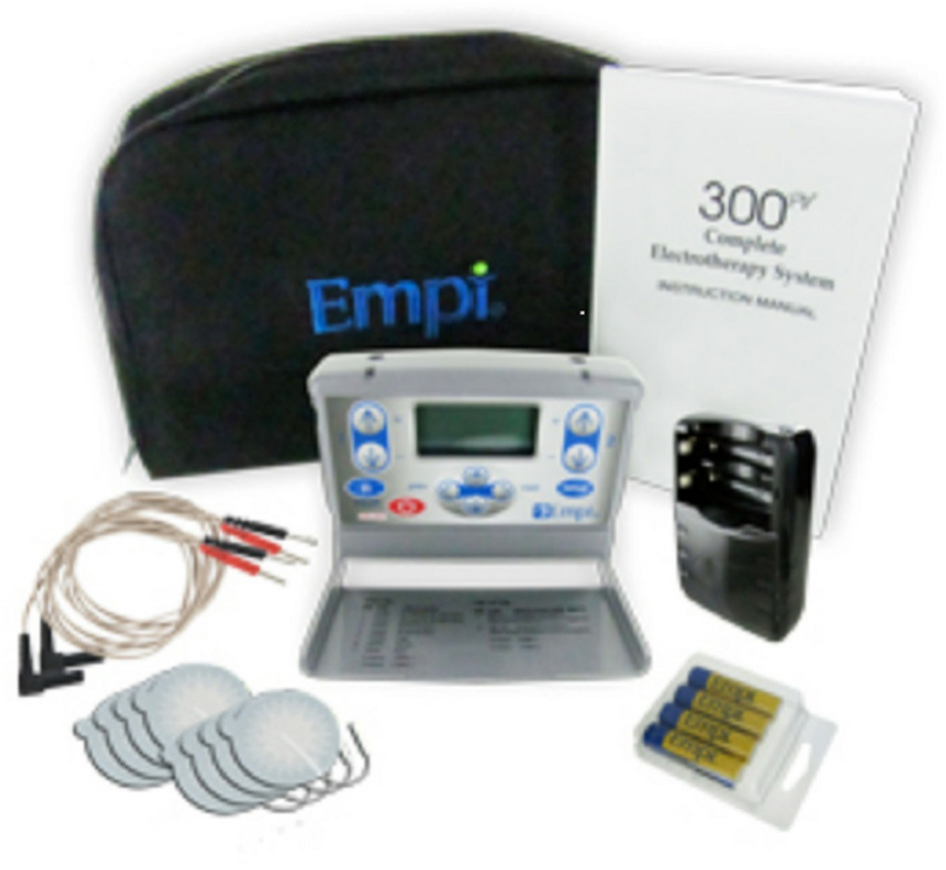
TENS, NMES (Empi 300PV unit).

**Figure 3:**
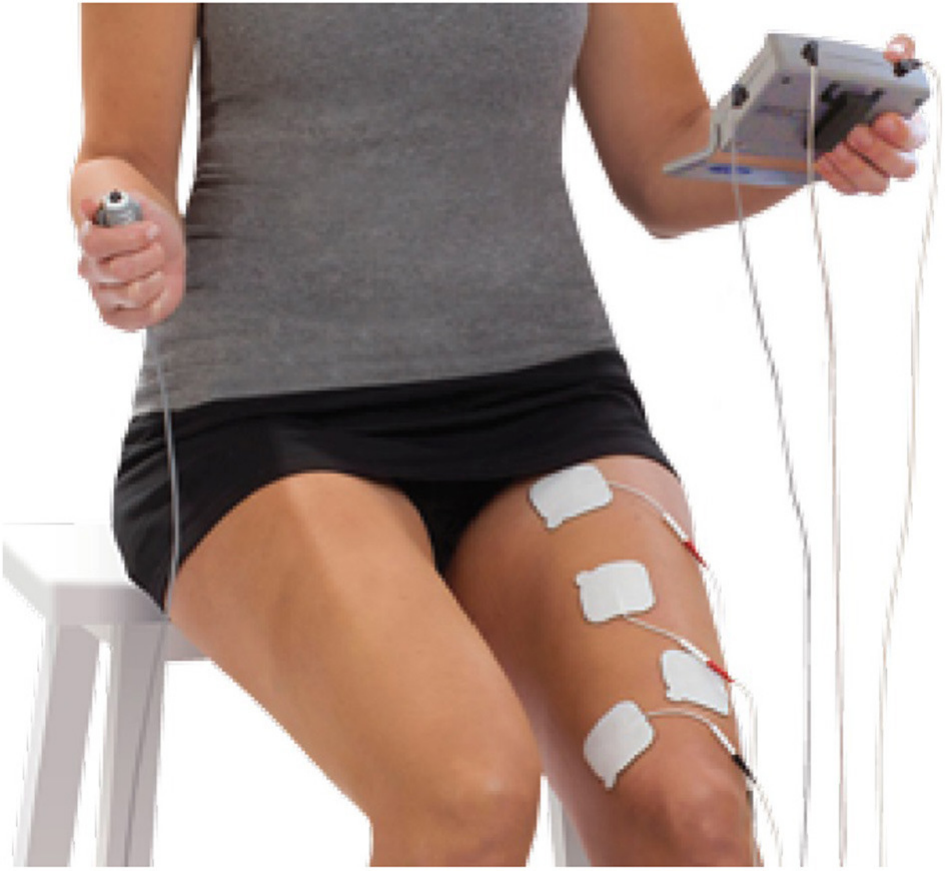
Demonstration of Empi 300PV unit.

**Figure 4:**
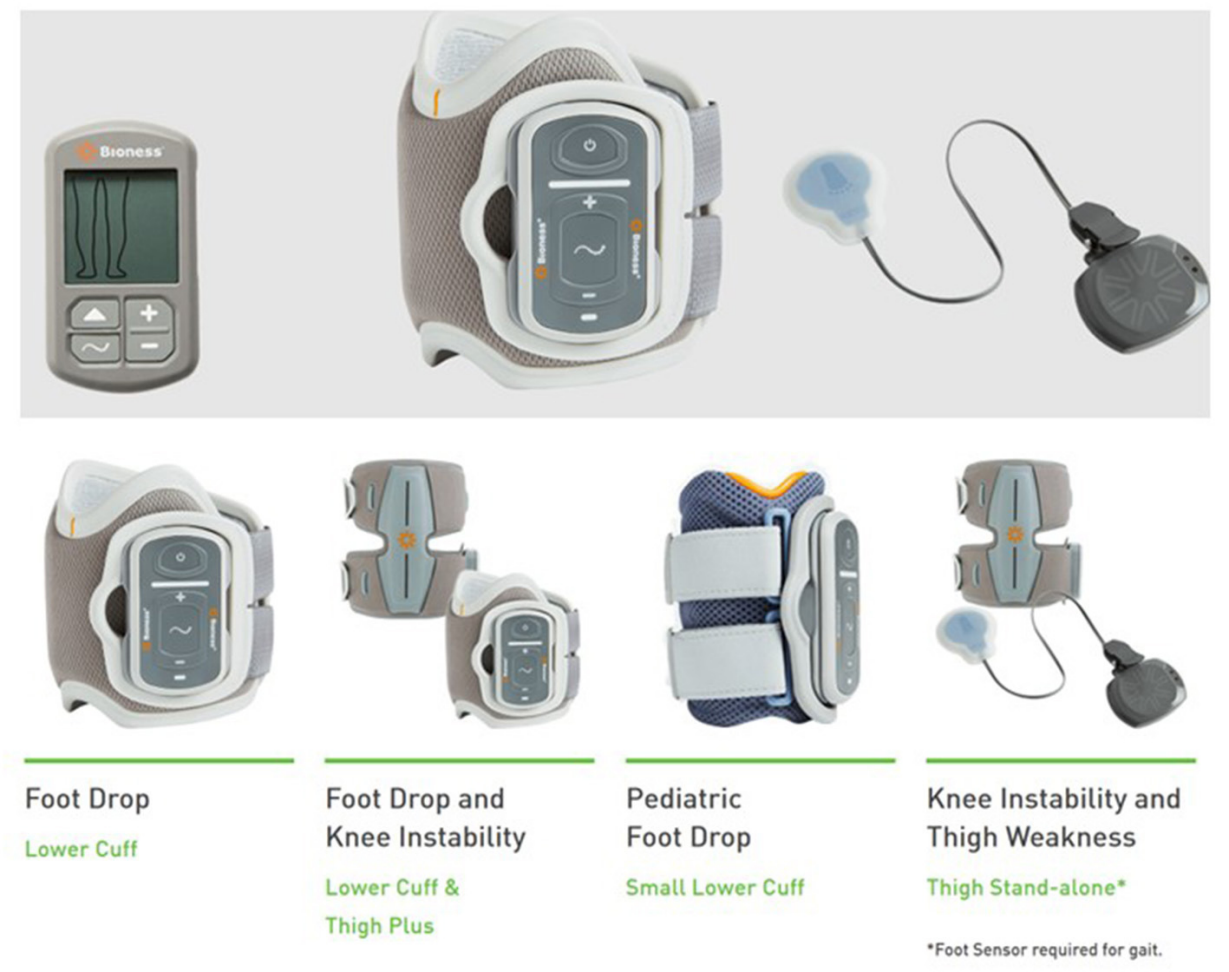
FES of foot (Bioness L300 Go).

**Figure 5:**
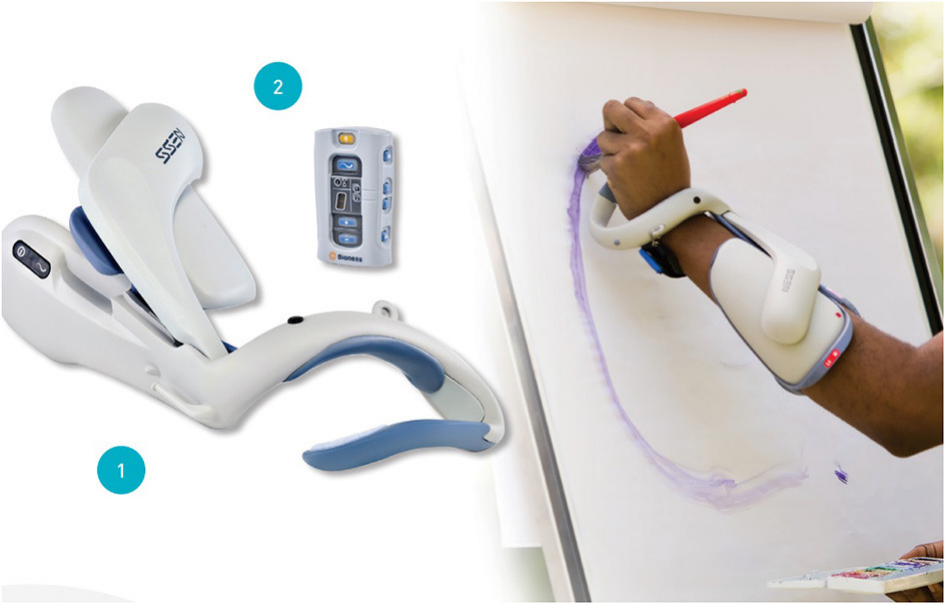
FES of hand (Bioness H200 wireless).

Since the 1980s multiple, animal studies have been conducted and have shown a positive effect of ES on peripheral nerve recovery. In a rat femoral nerve model, the use of 20 Hz continuous ES at the proximal end of the nerve reduced the axonal growth period from 10 to 3 weeks (13). ES that alters neuromuscular activity by electrical currents mainly includes neuromuscular electrical stimulation (NMES), transcutaneous electrical nerve stimulation (TENS), and functional electrical stimulation (FES). NMES usually produces muscle contraction at a frequency of 20–50 Hz and is used to improve patient function. TENS is usually used to relieve pain at a low frequency of 2–10 Hz or ultra-high frequency. Low-frequency TENS generally targets sensory nerves and does not produce visible muscle contraction. FES is a functional task, in which the target muscle is initially stimulated to generate movement, and the next step is to achieve the upper limb grasping the object or the lower limb walking. The current ES instrument is mostly small portable devices, such as 300 PV Empi, Bioness^®^ L300 Go, and Bioness^®^ H200 Wireless (Figures 2–5).

### 4.1. Neuromuscular electrical stimulation (NMES)

#### 4.1.1. Stimulation mode and system composition

When ES induces muscle contraction, H-reflex and M-wave waves are recorded by electromyography. (H-reflex is an action potential triggered by the current to afferent axons of LA fibers, which recruits low-threshold motor units; When the current intensity gradually increases, the motor axon is activated to produce a motor response called M wave.) This method of muscle activation, when the strength of the current applied by electrodes placed on the muscle or nerve causes muscle contraction, is referred to as NMES. NMES is commonly used to restore skeletal muscle mass and function in patients of PNI and is also applied to activate the nervous system in healthy individuals (14).

#### 4.1.2. Clinical application (nerve ES, direct muscle ES)

Some studies have investigated the effect of ES on nerve repair by directly connecting the two ends of the injured nerve to electrodes for brief ES when the skin is not sutured after nerve surgery. Gordon et al. (15) carried out a randomized controlled clinical trial (RCT) that assessed the post-surgical outcomes of acute brief low-frequency (20 Hz) ES. The researchers used ES (1 h; 20 HZ; 4–6 V; 0.1–0.8 ms) to improve neurological function in patients with severe carpal tunnel syndrome who had undergone surgery. It was shown that ES could promote axonal regeneration and accelerate muscle nerve regeneration (16). ES at 20 Hz for 1 h was also effective in cubital tunnel compression. One study involved Patients with severe cubital tunnel syndrome (CuTS), in which compression of the ulnar nerve in the cubital tunnel resulted in decreased neuromuscular function, and patients who underwent cubital tunnel release surgery were randomized in a 1:2 ratio into the control or PES groups. Patients in ES groups underwent ES (1 h; 20 HZ; balanced biphasic pulses; 30 V, 0.1 ms). Through three years of follow-up, using EMG signals to record motor unit number estimation (MUNE), grip, and key pinch strength, the study showed that, Brief-ES immediately after decompression surgery accelerated axon regeneration. Compared with the control group, postoperative MUNE function was better in the ES group, and grip and key pinch strength were also significantly improved. Patients who underwent surgery alone did not show any significant improvement in the number of MUNE, grip strength, or pinch strength. In one RCT (17), patients with complete digital nerve rupture received nerve repair and postoperative brief-ES (1 h; 20 HZ; balanced biphasic pulses; 30 V, 0.1–0.4 ms). At 6-month follow-up, patients in the ES group showed significant improvements in temperature discrimination, pressure detection, and spatial discrimination compared with controls.

Some researchers vary the duration of the stimulation cycle, the type of target muscle fiber, the width of the ES pulse, and the individualized setting of the ES intensity to explore the influence of different parameters on the effect of ES. In the studies of this paragraph, electrodes were attached to the proximal and distal ends of the muscle for direct muscle ES. Acaroz Candan S et al. (18) proved that NMES (100 Hz; symmetrical biphasic squared waveform; 400 μs) both a short stimulation period (SNMES 5 min × 4 sets) and a long stimulation period (LNMES 10 min × 2 sets) could improve quadriceps femoris function in the elderly, but there was no significant difference between the two groups. Toth et al. (19) used NMES (1 h; 50 Hz; symmetrical biphasic pulses; 400 μs) to intervention patients after anterior cruciate ligament injuries surgery, and the result showed that NMES could reduce the atrophy of slow-twitch fibers and fast-twitch fibers, and could maintain the muscle contraction strength and output power of slow-twitch fibers. The study by Stevens-Lapsley JE, et al. (20) demonstrated the ability to increase muscle strength and function by setting different personalized parameters according to the patient's maximum tolerance. Pinto Damo NL, et al. (21) used four different parameters of NMES, respectively narrow pulses (PC 200 μs), wide pulses (PC 500 μs), 500 μs phase duration, and low carrier frequency (KFAC 500: 1 kHz/Aussie current), and 200 μs phase duration and high carrier frequency (KFAC 200: 2.5 kHz/Russian current). The results concluded that KFAC and PC currents produced similar effects with the same phase duration. Currents with 500 μs induced higher muscle torque and efficiency, but patients felt more uncomfortable. Mani et al. (22) used both narrow pulses (0.26 ms) at 50 Hz and wide pulses (1 ms) at 100 Hz to improve mobility function in the elderly. The result showed both pulse methods improved lower limb strength and functional performance, but there were no significant differences between each other.

Moreover, excessive ES induces muscle fatigue and weakens the effect of improving nerve recovery. Vanderthommen et al. (23) suggested that brief intermittent ES was the best choice and high-frequency currents caused premature muscle fatigue. To avoid muscle fatigue, some scholars have done comparative studies between muscle ES and nerve ES. The result suggested that electrical nerve stimulation might be more comfortable and less to cause muscle fatigue than electrical muscle stimulation (24).

However, not all clinical applications of NMES are effective. Hyer et al. (25) used NMES to treat the calf muscles of patients after achilles tendon surgery. Neither muscle mass nor function was improved in the NMES group or the sham-stimulation group. Also in a double-blind randomized clinical trial, after traumatic peripheral nerve injuries with axonal damage and clinical impairment of two muscles. There was no significant difference between the ES group and the control group (26). Therefore, further studies are needed to determine the optimal parameters of ES. More clinical trials are expected to prove the effect of ES in the treatment of PNI.

#### 4.1.3. Animal models

In animal experiments, most scholars used the rat sciatic nerve transection and repair model to verify the effect and mechanism of NMES on nerve regeneration and prevention of muscle atrophy.

Compared with delayed ES after surgery, immediate ES is more conducive to neuromuscular recovery. It was reported that after sciatic nerve transection in rats, direct ES of the proximal sciatic nerve, immediate motor cortex stimulation (MCS) (60 Hz, 3–10 V, 200 μs) for 15 min and treatment for 2 weeks. The results showed that MCS was more effective than direct nerve stimulation in nerve regeneration and muscle nerve reinnervation, especially in the immediate postoperative period (27).

A similar effect was seen with 10 min of ES compared with 60 min. A study of sciatic nerve transection in mice was conducted and compared 10 min and 60 min ESs with pulsed current (28). The experimental results have shown that both settings facilitated motor neuron regeneration with increased axonal excitability, axonal myelin, and improved motor function. This finding has also been validated in tibial nerve transection repair, where as little as 10 min of ES could increase early axonal regeneration and produce similar benefits to 60 min stimulation (29).

ES may have different effects on different peripheral nerves. Researchers used ES (20 Hz, 3–4 V, 0.1 ms, 60 min) on the proximal femoral and facial nerves of rats after nerve cutting. The results showed that transient ES of the femoral nerve could promote nerve regeneration, but did not improve the facial nerve repair (30).

In the implantable ES experiment in rats, the contact area between the coil of the electrode and the nerve also had different effects on nerve regeneration. Some studies have compared three different nerve contact modes, which were point contact, 1/4 contact, and full coil contact. The results showed that the electrodes with point contact and 1/4 contact were more effective in promoting nerve regeneration and functional recovery (31).

#### 4.1.4. Mechanism

Peripheral nerve ES can be divided into direct muscle ES and nerve ES according to the electrode location. The mechanisms of the two kinds of ES have similarities and differences, which are summarized separately in terms of mechanism.

##### 4.1.4.1. Direct muscle ES

From animal studies, the mechanism of direct muscle ES on PNI may be high expression of myosin heavy chain (MHC) gene (32, 33). In experiments, surface electrodes were fixed to the skin of rats with nerve damage. The results showed that MHC expression in muscle was increased after ES, which promoted muscle strength recovery compared with the control group. A clinical trial has also verified that ES may achieve its effect by increasing MHC gene expression (34).

In addition, ES can also lead to an increase in light chain 3B-II (LC3-II) autophagy level. In one research, the sciatic nerve of rats was transected, and the proximal and distal parts of the nerve were repaired. The rats received ES (100 Hz, 200 μs, stimulation 5 s and intermittent 10 s, 30 min/day) for 2 weeks. The outcome measurements had the sciatic function index, the structure of muscle fiber, and the growth of nerve axons. The results have shown that the rehabilitation plus ES group was better than the control group. Among them, studies have observed that ES could increase LC3-II whose level represented the degree of autophagy. After adding an autophagy inhibitor, the effect of ES was attenuated. More powerful evidence was provided (35).

Moreover, ES can increase beneficial M2 macrophage, allowing faster nerve repair (36). In a clinical trial, ES combined with protein intake increased pro-inflammatory-like macrophages, which could activate the early step of muscle regeneration and accelerate collagen synthesis. ES might cause muscle damage, thereby accumulating inflammatory macrophages. Pro-inflammatory-like macrophages were decreased with aging, and the data from this study suggested that the increase of macrophages might be a positive adaptive response, and muscle loading alleviated muscle atrophy during the cessation of ES (36).

##### 4.1.4.2. Nerve ES

**Figure 6:**
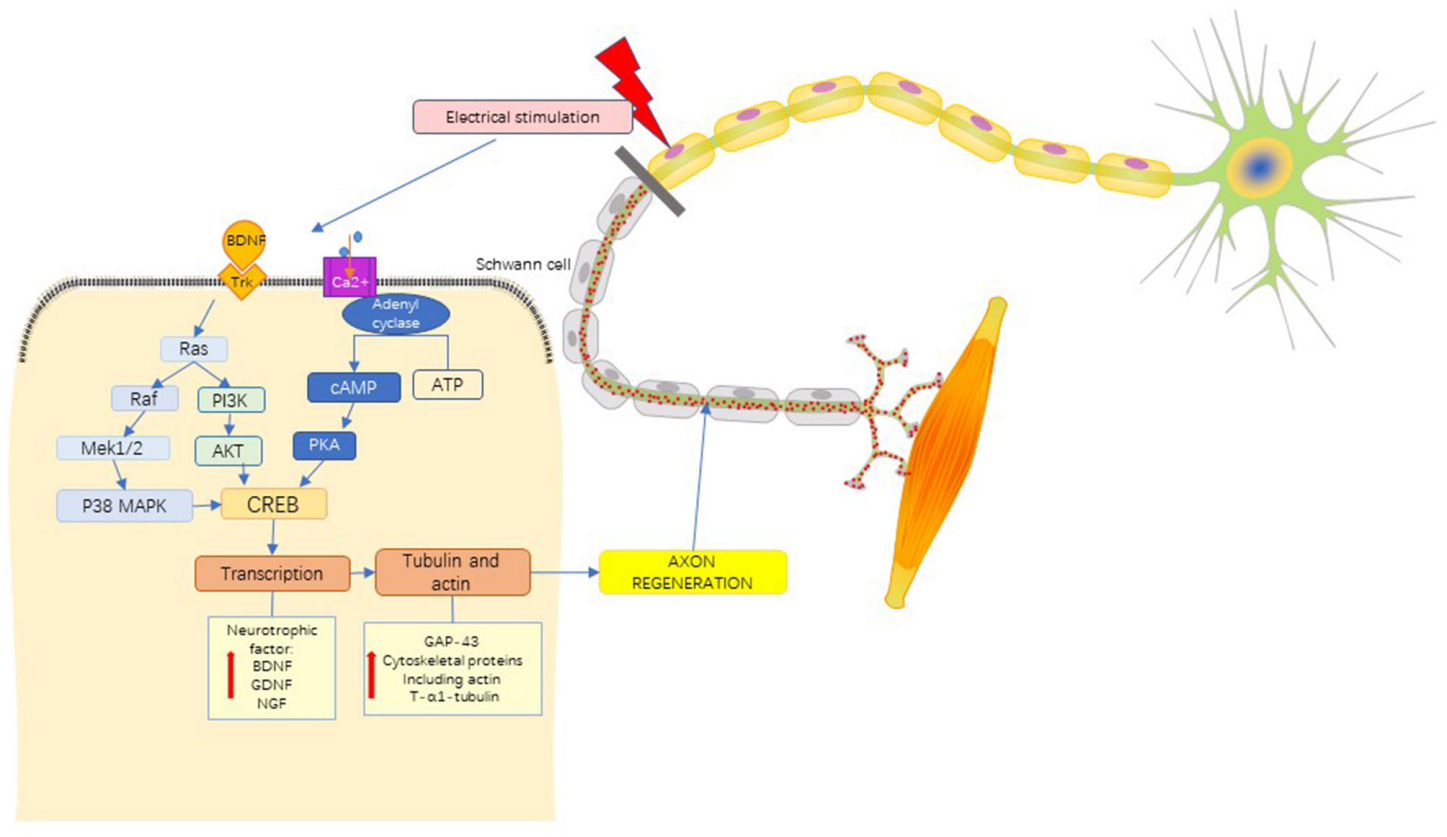
Mechanistic pathways of ES in the treatment of PNI. brain-derived neurotrophic factor, BDNF; Tyrosine kinase receptor, Trk; mitogen-activated proteinkinase kinase 1/2, MEK 1/2; P38 mitogen activated protein kinase, P38 MAPK; phosphoinositide 3-Kinase, PI3K; protein Kinase B, PKB(AKT); adenosine triphosphate, ATP; cyclic adenosine monophosphate, cAMP; protein kinase A, PKA; cAMP-response element binding protein, CREB; glial cell line-derived neurotrophic factor, GDNF; nerve growth factor, NGF; growth associated protein-43, GAP-43. See the text in the section “4.1.4 Mechanism” for a detailed description.

Previous studies have suggested that the effect of nerve ES is achieved by the up-regulation expression of brain-derived neurotrophic factor (BDNF) (37–39), glial cell line-derived neurotrophic factor (GDNF) (40), Tyrosine Kinase receptor B (TrkB) (41, 42) and Increasing of adenosine monophosphate (cAMP) (43). ES can cause Ca^2+^ influx, and the increase of Ca^2+^ in nerve cells induces the up-regulation of BDNF and TrkB expression (44). Overexpression of BDNF can inhibit phosphodiesterase activity, resulting in a sustained increase in cAMP levels (38, 45). Cytoskeleton formation is accelerated by activation of cAMP-response element binding protein (CREB), upregulation of RAGs such as T-α-1 tubulin and growth-associated protein-43 (GAP-43) expression (46), and inhibition of Rho (47). In addition, trkB-stimulated Ras activated CREB through P38 mitogen-activated protein kinase (P38 MAPK) pathway (18) and activation of kinases phosphatidylinositol 3-kinase/Akt (PI3K/Akt) pathway (48) to enhance the effect of ES on peripheral nerve regeneration (Figure 6).

The repair of nerve injury was delayed for different time durations (2, 4, 12, and 24 weeks), brief depolarizing ES (20 Hz, 3 V, 20 min) was applied to rats that were bridged with a hollow nerve conduit after sciatic nerve transection. The results have shown that the diameter and number of regenerated axons, myelin sheath thickness, and the number of the motor and sensory neurons significantly increased in delayed nerve injury repair. The brief ES also increased the expression of BDNF, which accelerated axon regeneration by BDNF-mediated neurotrophin signaling (37).

ES to promote regeneration of PNI may be through the increase of neurotrophic factors and cAMP. One study involved patients with severe carpal tunnel syndrome, the results proved that ES affected nerve recovery. To further elucidate the underlying mechanisms, they also injected agonists of cAMP into rats after femoral nerve transection surgery. Similar effects were achieved, which demonstrated the mechanism by which ES accelerated axonal regeneration (49).

Formation of synapses and the sustaining of motor neuron's cell body including vesicle glutamate transporters 1 (VGLUT 1) (50, 51). Park et al. (50) used mice with sciatic nerve transection to show that both single and repeated ES increased the direct muscle responses. Mice that received a single ES showed a progressive increase in muscle contraction amplitude during recovery. Only in repeated treatment mice, cell bodies of excitatory and inhibitory synaptic contacts were sustained, including VGLUT 1. The maintenance of VGLUT1+ inputs onto motoneurons might be related to the expression of BDNF and its trkB receptor. Moreover, the H-reflex became twice its pre-injury level after repeated ES, which indicated that repeated ES could preserve muscle reflex by sustaining of VGLUT 1 (50).

In addition to nerve cells, Schwann cells (SC) are also affected by ES. In the animal model of tibial nerve transection, 1-time brief ES during surgery was used to improve the axonal regeneration of the transplanted nerve. The results observed that ES could convert M1 macrophages, which cause inflammation, into M2 macrophages, which are beneficial for repair, thereby rapidly removing myelin debris and improving neurological function (29, 52).

Some scholars believed that ES could redistribute blood flow to active muscles and meet muscle metabolic demands, which encouraged muscle contraction (53, 54). In animal experiments, nerve ES can increase the structural changes of capillaries in rats with nerve injury, thereby increasing blood flow, providing sufficient oxygen, and accelerating peripheral nerve regeneration (53). Especially at the current intensity of 10 mA and above, the effect of ES on accelerating the recovery of PNI was more significant (54).

### 4.2. Transcutaneous electrical nerve stimulation (TENS)

#### 4.2.1. Stimulation mode and system composition

ES that uses a small current to activate sensory axons without triggering muscle contraction is called TENS. It is often used to treat pain, spasticity, and urinary incontinence (55). The commonly used frequency in clinical practice is 1–150 Hz (56). In rehabilitation, TENS are used to improve sensory feedback and adjust neural network pathways. Sensory feedback plays an important role in completing an action. When the motor nerve is damaged and unable to conduct, the sensory nerve will temporarily innervate the muscle to prevent muscle atrophy until the motor nerve recovers. A concept is termed chipmaker protection or chipmaker “babysitting” (57). TENS can improve sensory feedback which is important for the movement of the body. Pain is one of the sensory disorders that patients complain about most obviously and affect their daily life most, and it is also the most subjective sensory disorder (56). Therefore, there is more corresponding research in this area.

#### 4.2.2. Clinical application (ultra-high, high, and low-frequency; implantable)

TENS has high and low-frequency modes. Low-frequency TENS is defined as the delivery of pulsed currents of 10 Hz or less (burst mode of TENS). High-frequency TENS is used to describe frequencies more than 10 Hz to the maximum setting on TENS devices, typically 150–200 Hz (58).

Bergeron-Vézina et al. (59) have compared the improvement effect of low-frequency TENS (110 Hz; 200 ms) and high-frequency TENS (unmodulated 5-kHz; 200 ms) on neurological function. Outcomes were no differences in mechanical pain threshold (MPT), heat pain threshold (HPT), tactile threshold (TT), and peripheral nerve conduction (PNC). However, patients felt more comfortable with Unmodulated 5-kHz currents. In an RCT (60), both high-frequency (100 Hz, 60 us) and low-frequency (3 Hz, 400 us) TENS improved pain in young adults, but there was no effect on the pain perception threshold of 15 elderly people (67 years old), reflecting that the elderly are less sensitive to TENS than young people.

One paper has shown that TENS with the patient's tolerance intensity can also improve pain. A meta-analysis of RCT has shown that non-invasive TENS could reduce pain by 30%, with the recommended intensity being “strong but comfortable” as optimal, and “mild”, “faint” or “barely perceptible” as suboptimal (58). In one study, TENS could improve 72 patients of non-cancer pain including peripheral nerve pain, which had affected life severely lasting more than 3 months. The ES parameter was the optimal frequency provided by the machine, and the intensity was standardized on patient tolerance. With pain improvement in 40% of patients at 6 months of treatment (61).

Some researchers have used ultra-high-frequency TENS to treat pain. The frequency was higher than 10 kHz and the treatment had a significant effect (62, 63). In a trial, 20kHz of percutaneous high-frequency alternating current (HFAC) was applied to the ulnar and median nerves of volunteers for 20 min. The current intensity from the beginning to the end of the stimulation ranged from 44.2 mA to an average final intensity of 85.0 mA. Outcome measurements had maximal handgrip strength, mechanical threshold, maximal handgrip strength (MHS), mechanical detection threshold (MDT), and pressure pain threshold (PPT). The results have shown that 1. The 20 kHz stimulation showed the lower MHS during the stimulation at the 15 min and 20 min when compared to the sham-stimulation group 2. The 20 kHz stimulation resulted in a slight increase in MDT at 15 min when compared to the sham-stimulation group 3. No effects were shown for PPT. The conclusion was that HFAC caused a partial block of nerve activity, which might be a therapeutic approach to the hyperactivity of the nervous system. Chronic pain can also be alleviated by nerve conduction block, but the optimal parameters are unknown (62). A double-blind trial used invasive ES at 10 kHz for chronic pain in 58 spinal cord (SCS) and 11 peripheral nerve (PNS) patients. Both groups showed a reduction of pain and disability after 3–6 months of stimulation, but PNS relieved pain to a greater extent than SCS. In patients with PNS, the effect of pain relief after stimulation was maintained for 12 h. There was no change in pain 2 h after stimulation in PNS patients, but the pain was significantly reduced after 4 h. In summary, 10 kHz ES performed for at least 4 h could reduce pain in patients with PNS (63).

For TENS and HFAC in improving pain which one is more effective, one study has shown that compared with the sham-stimulation group, 40 min of TENS can significantly inhibit the amplitude and lengthen the latency of the soleus H-reflex. TENS and HFAC at a frequency of 10 kHz had the effect of regulating the soleus H-reflex, but there was no meaningful difference between the two groups (62).

At the same time, a lot of clinical trials have shown that implanted TENS had a significant improvement in pain after PNI. Implantable TENS is an effective method for peripheral nerve pain caused by upper limb trauma. This technology in the armpit implantation of a nerve stimulator and four electrodes were placed on the affected sensory nerve branches (mainly brachial plexus, median nerve, and radial nerve). According to the characteristics of each patient set the stimulation parameters. Stimulation was an action for 24 h after nerve repair surgery. The results have shown that implantable TENS could significantly relieve and prevent intractable pain caused by PNI. Implantable TENS preserved the structure of neuroanatomy, which was an effective and feasible treatment method (64). Similarly, implanted TENS (12 Hz, 20 mA, 20–200 μs) was used to treat refractory pain caused by subacromial impingement syndrome (SIS). The electrodes of a small ES wearable device were implanted into the end of the axillary nerve and the deltoid muscle, and the surface electrode was attached to the skin. Longitudinal analysis has shown a significant reduction in pain, which was most pronounced at 5, 8, and 16 weeks after treatment (65). In an RCT, electrodes were implanted at the affected shoulder axillary nerve in patients with shoulder impingement syndrome (12 Hz, 0.2–30 mA, 10–200 μs). The results have shown that axillary nerve ES had a good effect on chronic shoulder pain (66).

Some studies have developed an intermediate frequency alternating current (10 kHz, 0.3 s), and surface electrodes were applied to the median nerve. The results have shown that this intermediate frequency alternating current could be used to inhibit undesired sensory and motor activities and accelerate nerve repair (67).

However, there are conflicting results compared to previous studies. In a study to treat decreased shoulder strength after exercise-induced acute muscle pain. TENS (85 Hz, 0–80 mA, 75 μs) was compared with the sham-stimulation group, and electrodes were placed around the rotator cuff. The results have shown that the two groups had no significant effect on pain relief, but had a Nocebo effect on shoulder muscle strength. This study suggested that the application of TENS was complex, and it was necessary to separate the psychological effects and sensory mechanisms of TENS to determine the outcomes of patients with pain (68).

One study has shown that the use of TENS during exercise in elderly patients with chronic pain could not improve the prognosis of patients, but it had a transient analgesic effect during exercise and was well tolerated by the elderly (69).

#### 4.2.3. Animal model

A previous study used rats with sciatic nerve crush to verify the effect of ES, low-frequency ES (LFES 5 Hz), and high-frequency ES (HFES 100 Hz) in the early stage (at that time after injury) and late stage (7 days after injury). The results of the experiment were evaluated by motor function recovery score, thermal hyperalgesia test, gait analysis, and somatosensory cortex evoked potential. The results showed that immediate HFES significantly improved motor function but increased the susceptibility to neuropathic pain. Compared with LFES, HFES can increase the growth of nerve myelin sheath in both early and late stages. Late stage of HFES increased nerve regeneration without aggravating neuropathic pain in nerve crush injury. Moreover, cell experiments were also performed, in which the dorsal root ganglion cells of rats received ES at 5 Hz and 100 Hz for 30 min at 50 mA. The results of cell experiments, ES could activate dorsal root ganglion cells to express inflammatory cytokines, such as TNF-α, synapsin, and NGF (70).

In an animal experiment, 60 rats were divided into a sham operation control group (SHAM), a sciatic nerve denervation group (DN), and sciatic nerve denervation plus ES (DN-SM). Surface ES (2 Hz, 25 V, 1 mA, 300 ms, 10 min/day) was performed for 28 days with one electrode attached to the achilles tendon and the other to the popliteal fossa. Proteomics, transcriptomics, bioinformatics, and skeletal muscle function analysis were used to observe the molecular expression changes induced by ES. The results showed that the DN-SM group was better than the DN group in terms of muscle mass, muscle fiber diameter, and contractile properties. At the molecular cellular level, the FoxO and p53 signaling pathways are important in structural protection by bioinformatics analysis. Anti-apoptosis proteins (KCNA7, KCNJ11) were down-regulated after denervation but up-regulated after ES. Muscle fiber type-related proteins (TNNI1, TNNT1, ACTN2), myosin light chain kinase 2 and myomesin 2, fibrosis-associated proteins (POSTN, COL1A1, COL1A2, COL6A1, COL6A2, COL6A3, FN1, and LUM) were all increased after denervation but decreased by ES. All these results explained the mechanism of ES in promoting nerve regeneration (71).

#### 4.2.4. Mechanism

The mechanism of TENS to relieve pain and promote nerve regeneration may be that (1) TENS can stimulate low-threshold skin afferents to inhibit the positive transmission of nociceptive information in the central nervous system, thereby relieving pain (also known as, segmental modulation). In addition, TENS can stimulate the small-diameter afferent pathway to activate the descending pain inhibition pathway or block the afferent activity of peripheral neurons, forming a “busy line” effect (72). (2) It has been confirmed in the human body that ES can block nerve conduction and reduce H-reflex excitability, thereby improving the situation of abnormal increase in H-reflex caused by nerve overactivities, such as spasticity and pain (73). (3) Powerful evidence confirmed that from the aspects of hemodynamics, increasing blood flow to the ES can restore nourishment. In a study comparing TENS and NMES influence on the hemodynamics of the gastrocnemius muscle, the results showed that ES can increase muscle hemodynamics. Compared to the NMES, TENS can increase blood flow even more (74). (4) Animal experiments have shown that ES can activate nerve cells to express inflammatory cytokines. For example, the FoxO and p53 signaling pathways were activated, and muscle-related structural proteins were increased (71).

### 4.3. Functional electrical stimulation (FES)

#### 4.3.1. Stimulation mode

FES is the ES of muscles or nerves to provide functional improvement. Applications of FES include restoration of upper limb functions, such as stretching and grasping and lower limb functions, such as standing, balance, posture, and gait. There are three types of stimulation methods called fully implanted, percutaneous stimulation, and surface stimulation (75). For treatment, surface stimulation is preferable because it does not invade the body. Current surface electrodes use biocompatible gels that provide stability on the skin and uniform current distribution on the electrode surface. Conventional surface electrodes are suitable for innervating large muscles close to the skin (76).

#### 4.3.2. Clinical application (activity function, muscle fatigue, latest technology)

The FES mainly focuses on the walking function of the lower limbs and the finger-grasping function of the upper limbs. A surface FES of the tibial nerve in healthy participants showed that FES activated both thigh and calf muscle contraction. During walking, contraction of the thigh and calf muscles plays an important role in gait. In the experiment, the anode (50 Hz, 300 ms) was fixed on the calf gastrocnemius muscle, and the medial gastrocnemius muscle, tibialis anterior, semitendinosus, and rectus femoris were recorded by the electromyography (EMG). This study has shown that FES had a significant effect on the contraction and activation patterns of muscles (77).

Using FES during walking, 30 min of stimulation could increase the half-maximum peak-to-peak motor evoked potential (MEPh) of the tibial nerve, and the effect lasted for at least 30 min. In addition, the results showed that only the combination of FES and exercise could lead to an increase in corticospinal excitability without cortical inhibition and further promote the afferent of the central nervous system (78).

It has been performed that FES on the ulnar nerve and median nerve was effective for upper limb motor function. A non-transcutaneous FES was performed on the proximal ulnar and median nerve bundle. Individual finger and joint grasping movements were observed using 24 fingers movement to quantify hand grasping patterns. The results have shown that this stimulation technique was able to stimulate individual and coordinated movements. The grasping pattern was different depending on the location of the stimulation. In the future, it can be used for the treatment of grasping with weak fingers (79). A similar trial used 30 Hz ES with three anode electrodes placed in the proximal segment of the inferior radial nerve to activate and control different finger and wrist extension movements (80).

ES of the radial and median nerves in eight tetraplegic patients was able to provide useful grasping movements. A multi-contact cuff electrode (25 Hz, 250 μs) was implanted around the median or radial nerve 5 cm above the elbow of the subjects. Through evaluating the flexion and extension of the thumb, finger, wrist, and functional movements, the results have shown that this minimally invasive ES could effectively restore the patient's grip function. Future studies using two microelectrodes to activate more muscle activity are expected (81).

In addition to the coordination of various parameters to achieve the purpose of stimulating muscle and nerve recovery, excessive muscle fatigue caused by FES will reduce the effect. To alleviate muscle fatigue, some studies have further improved the stimulation parameters. Some scholars have used ultrasonic echogenicity as an evaluation index of FES-induced muscle fatigue, and there was a strong linear relationship between ultrasonic echogenicity and muscle fatigue level. Muscle-in-the-loop FES controllers considering muscle fatigue are helpful to produce better stimulation effects (82).

Researchers have used multiple electrodes to activate a larger volume of muscle to reduce muscle fatigue. The results have proved that ES with four intramuscular electrodes was more conducive to muscle contraction and endurance than ES with a single intramuscular electrode. In addition, it was further demonstrated that muscle fatigue might be caused by single FES not activating the intact motor synapses of the muscle (83). For muscle fatigue or damage caused by prolonged stimulation, a cross-sectional study used ES with different parameters to treat 24 cases of denervated extensor digitorum communis muscle and 24 cases of denervated tibialis anterior muscle. Different combinations of pulse duration and polarity were evaluated using triangular pulses, with current increasing from 0.1 mA and a frequency of 1 Hz. The results have shown that the triangular current with a duration of 200 ms and cathode polarity had a better effect on the denervated tibialis anterior muscle, which was statistically significant (84).

One study used FES at 10, 35, and 50 Hz on two muscles (vastus lateralis, VL; abductor pollicis brevis, APB) with different proportions of fast fibers and slow fibers. The results have shown that, In high-frequency stimulation, VL with more fast muscle fibers was tired faster than APB with more slow muscle fibers, and the treatment time should not exceed 14–16 min (85).

Jaramillo Cienfuegos et al. (86) have used a proportional integral (PI) controller to achieve classical and adaptive control of FES for isolated skeletal muscle contraction, which provided the best closed-loop performance for contraction speed and anti-interference. Future research should use algorithms to control FES muscle contraction, focusing on different stimulation sites. On the premise of avoiding muscle fatigue caused by stimulation as much as possible, the best stimulation effect was evaluated by the combination of each stimulus parameter. We summarized the various ES parameters applied to PNI (Supplementary Table 1).

#### 4.3.3. Animal models

Animal experiments have shown that ES could significantly accelerate the regeneration of injured peripheral nerves. Some scholars have conducted animal experiments to prove that ES could promote the recovery of animal nerve function and increase the strength of muscle contraction (87). In the experiment, FES was used to activate the maximum muscle contraction force in the anterior deltoid muscle of monkeys, showing that the distributed dual electrode could generate an additional 50% contraction force compared with the single electrode, which improved the effect of FES.

In the experiment of horses with recurrent laryngeal nerve injury, FES was implanted into the ipsilateral posterior cricoarytenoid muscle (PCA) after 20 weeks of ES, which improved muscle strength and laryngeal function (88).

An implantable microelectrode array (MEA) belongs to the scope of FES, which is used to prevent muscle atrophy and acetylcholine receptor degradation during nerve regeneration after PNI. In one study (89), the tibial nerves of rats were cut, and MEA was placed on the surface of the biceps femoris muscle. The results have shown that the atrophy degree of muscle fiber cross-section in MEA-mediated FES rats was less than that in control rats, and the area of acetylcholine receptor was significantly increased.

#### 4.3.4. Mechanism

At present, the mechanism hypothesis of FES promoting nerve regeneration and muscle contraction after PNI is as follows: (1) It increases the blood flow of the stimulated muscle capillaries and the flux of red blood cells (90). (2) FES is mainly applied at the motor point (motor point stimulation, MPS). Some studies have explored the neural pathways activated by MPS, and the results have shown that MPS did not induce H reflex or LA sensory nerve activation, but it could induce H reflex inhibition of stimulated muscles and skin inhibition (91).

## 5. Combined application of ES and other rehabilitation methods (exercise, phototherapy, etc.)

ES plays a role in promoting the recovery of PNI. Some studies have combined ES with other rehabilitation methods to achieve better therapeutic effects. The main combined rehabilitation methods include exercise training (40, 50, 92), phototherapy, magnetic stimulation, cryotherapy, etc.

ES combined with exercise can more strongly promote peripheral axon regeneration and relieve pain after PNI (40, 50). An RCT has shown that physical therapy exercise training combined with TENS was more effective in treating nerve pain in men (92). In a controlled study, low-level laser (continuous wave: 15 mW, 632.8 nm; Pulsed: 9.4 W, 904 nm) plus microamperes TENS had a significant effect compared with the control group in the treatment of carpal tunnel syndrome pain. And the improvement effect could be maintained for 1–3 years (93). The combination of low-frequency transcutaneous magnetic stimulation and ES in the treatment of PNI could counteract the slowing effect of TENS on the fast conduction fibers, and regulate the slow conduction of pain afferent fibers, resulting in better analgesic effect (94). Some scholars have combined cryotherapy with burst TENS to effectively improve the pain threshold of participants, and the effect of the combination was better, but the combination of cryotherapy and ordinary TENS had no improvement (95). Some researchers have also obtained negative results. Compared with the use of NMES or motor images alone, the combination of NMES and motor images did not achieve better results (96).

Similar studies in animal experiments have shown that ES could achieve better results when combined with exercise training, magnetic stimulation, and stem cell therapy. After sciatic nerve transection repair in adult rats, the rats were divided into 4 groups, the immediate ES after injury (ESA), delayed ES, ES+ exercise, and exercise groups. The results have shown that compared with the control group and the rats receiving delayed ES, ESA with or without exercise group improved muscle reinnervation and increased the number of regenerated myelin axons. ESA combined with exercise significantly improved muscle reinnervation at an early stage (97). In an animal experiment, electrical muscle stimulation prevented muscle mass loss. Combined with exercise, twitch characteristics, fatigue index, mechanical sensitivity, and mechanosensitivity could be further recovered (98). PNI would enhance the transmission of pain fibers and lead to chronic post-traumatic pain. Animal experiments have shown that magnetic stimulation and ES could promote the recovery of PNI and allow a tolerance of high-intensity output (99). The combined effect of ES and stem cell therapy could better promote nerve regeneration and improve functional recovery after sciatic nerve transection in rats. ES was able to up-regulate the expression of neurotrophic factors (BDNF, NTF-3) and increase the expression of neurotrophic factor receptor (Trk) in human neural progenitor cells (hNPC). Thus, it was capable of promoting angiogenesis, axon dendrite growth, myelin sheath thickening, and accelerate peripheral nerve regeneration and functional recovery (39).

## 6. Limitations of clinical application

In conclusion, ES can accelerate the recovery of body function after PNI, but ES still has the following problems to be solved, (1) What are the changes in brain activity induced by ES. (2) Optimal ES parameters are expected. (3) Further clinical applications provide more authority evidence to verify the effectiveness of ES. (4) Solving the side effects of ES.

Most importantly, the changes in the central nervous system after PNI are not well understood, which is the basis for determining the appropriate method of ES and finding new methods. Although there are many positive results of ES, there are also some doubts about perceptions. For example, some studies have shown that ES could aggravate muscle fiber atrophy, reduce muscle excitability, and inhibit peripheral nerve regeneration (100).

## 7. Conclusion

The advantage of NMES can activate type II fibers that are most affected by aging and responsible for the decline in functional activity (18). The disadvantage of high-frequency continuous NMES is easy to causes muscle fatigue. PNS patients have higher comfort than NMES patients.

TENS has a good effect on the treatment of stubborn pain caused by PNI, and non-invasive or minimally invasive TENS is easy to be accepted by patients. The disadvantages of TENS are that the effect is short, and the elderly are less effective than the young because of their decreased sensitivity.

The advantage of FES is that targeted stimulation of a nerve or muscle can produce stable effects for different motor functions. However, it is uncomfortable to wear an ES instrument for a long time. Prolonged FES may cause muscle fatigue and decrease responsiveness.

With the number of patients with PNI increasing, more attention should be paid to the repair effect of peripheral nerves. This paper believes that ES can accelerate neurological recovery of PNI, effectively relieve pain and increase muscle mass and strength in patients, and it is necessary to select appropriate ES methods and parameters according to the actual situation of patients.

## Author contributions

LN: conceptualization, methodology, and validation. ZY: searching literatures and writing—original draft preparation. YZ: visualization and formal analysis. TZ: supervision and data curation. JW: software and validation. SL: writing—reviewing and editing. ZC: funding acquisition and supervision. All authors contributed to the article and approved the submitted version.
